# Quality of Life Measured Using the BODY-Q After Adolescent Gynecomastia Surgery: A Cross-Sectional Analysis

**DOI:** 10.1177/22925503241249753

**Published:** 2024-05-07

**Authors:** Marta Karpinski, Young Ji Tuen, Rebecca Courtemanche, Jugpal S. Arneja

**Affiliations:** 1 Division of Plastic Surgery, 37210 BC Children's Hospital, Vancouver, BC, Canada; 2 Faculty of Medicine, Division of Plastic Surgery, 8166 University of British Columbia, Vancouver, BC, Canada; 3 Sauder School of Business, University of British Columbia, Vancouver, BC, Canada

**Keywords:** gynecomastia, surgery, quality of life, personal satisfaction, Gynécomastie, chirurgie, qualité de vie, satisfaction personnelle

## Abstract

**Background:** Patient-reported outcome measures (PROMs) are increasingly used to seek feedback from patients. Knowledge gaps exist regarding outcomes in adolescents postgynecomastia surgery. This study assesses adolescent patients’ quality of life postgynecomastia surgery using a PROM specific for body contouring procedures, and identifies patient and treatment factors associated with quality of life scores. **Methods:** Adolescent patients undergoing surgical treatment for Simon grades I, IIa, or IIb gynecomastia from May 2009 to November 2022 at British Columbia Children's Hospital were invited to complete the Body Contouring Questionnaire (BODY-Q) *Satisfaction with Chest* and *Psychological Function* scales. Raw scores were Rasch transformed (scale 0-100), averaged, and compared to normative scores. Body mass index (BMI), Simon grade, postoperative complication, and revision procedure information were collected from medical charts and analyzed for associations with BODY-Q scores. **Results:** Thirty-seven of 76 patients (48.7%) completed the BODY-Q. At the time of survey completion, the median age of participants was 23.3 years, and the median BMI was 26.5. The median time from surgery to survey completion was 7 years. Median scores on the *Satisfaction with Chest* and *Psychological Function* scales were 70 and 71, respectively, which were significantly higher than normative scores of unaffected males on the same scales (*P* = .0004 and *P* = .0014, respectively). Undergoing a revision procedure was associated with significantly worse satisfaction with chest appearance (*P *= .045). **Conclusion:** Patients who underwent gynecomastia surgery report better satisfaction with chest appearance and psychological function, as measured by the BODY-Q, compared to unaffected males of similar age and BMI. These results may play an important role in destigmatizing gynecomastia surgery for adolescents.

## Résumé

**Contexte :** Les mesures des résultats décrits par les patients (PROM) sont de plus en plus utilisées pour obtenir un retour d’information de la part des patients. Il existe des lacunes dans les connaissances concernant les résultats de la chirurgie postgynécomastie chez les adolescents. Cette étude évalue la qualité de vie après chirurgie pour gynécomastie chez des patients adolescents au moyen d’une méthode spécifique de PROM pour les procédures de remodelage corporel et identifie les facteurs liés au patient et au traitement associés aux cotes de qualité de vie. **Méthodes :** Les adolescents ayant subi un traitement chirurgical de gynécomastie de stade 1, 2a ou 2b de Simon entre mai 2009 et novembre 2022 au BC Children’s Hospital ont été invités à remplir le questionnaire de BODY-Q avec des échelles de satisfaction sur l’aspect du thorax et sur les fonctions psychologiques. Les cotes brutes ont été transformées à l’aide du modèle de Rash (échelle de 0 à 100), moyennées et comparées à des cotes normatives. De l’information sur l’IMC, le stade de Simon, les complications postopératoires et les procédures de révision a été collectée à partir des dossiers médicaux et analysée à la recherche d’associations avec les cotes BODY-Q. **Résultats :** Trente-sept patients sur 76 (48.7 %) ont rempli le BODY-Q. Au moment de la fin de l’enquête, l’âge médian des participants était de 23.3 ans et l’IMC médian était de 26.3. La durée médiane écoulée entre la chirurgie et la fin de l’enquête était de 7 ans. Les cotes médianes sur les échelles de satisfaction thoracique et des fonctions psychologiques étaient respectivement de 70 et 71, soit significativement supérieures aux cotes normatives des individus masculins non concernés sur les mêmes échelles (respectivement *P* = .0004 et *P* = .0014). Subir une procédure de révision a été associé à un niveau de satisfaction de l’aspect du thorax significativement plus mauvais (*P* = .045). **Conclusion :** Les patients ayant subi une chirurgie pour gynécomastie ont déclaré une plus grande satisfaction de l’aspect de leur thorax et une meilleure fonction psychologique, mesurée par le BODY-Q que les sujets masculins non affectés, de même âge et même IMC. Ces résultats jouent un rôle important pour déstigmatiser la chirurgie de la gynécomastie pour les adolescents.

## Introduction

Adolescent gynecomastia is typically a benign condition that is self-limited in 75% to 90% of cases, and usually resolves over a 1- to 3-year period without the need for intervention.^1^ Therefore, in the absence of history, signs, and symptoms suggestive of an underlying pathology or secondary cause, monitoring and reassurance for patients and their families are the mainstay of early management.^2^ Surgical treatment is typically reserved for gynecomastia cases that have persisted beyond 2 years.^2,3^

However, gynecomastia during adolescence, a period of increased awareness of one's self-image and sexual identity, has raised important questions about the psychological impact of gender-incongruent development, and the role of earlier surgical intervention in the context of significant psychosocial distress secondary to the condition.^2,4,5^ To date, several studies have demonstrated the detrimental psychological impact of gynecomastia during adolescence, which results in impairments in social functioning, mental health problems such as depression and anxiety, as well as diminished self-esteem.^4,5^ In an attempt to conceal their chest, some patients engage in coping behaviors such as wearing multiple layers of clothing, hunching their shoulders, and binding their chest with household items, which in turn may cause discomfort, pain, and even injury.^5^

Surgery for gynecomastia has the potential to significantly improve patients’ quality of life. Nuzzi et al^6^ previously showed that adolescent patients with gynecomastia scored significantly worse on the Rosenberg Self-Esteem Scale (RSES) and in several domains of the Short-Form 36 (SF-36) compared to unaffected male controls. Postoperatively, RSES and SF-36 scores significantly improved for gynecomastia patients and, in fact, were comparable to those of unaffected controls.^6^ Similar improvements in psychosocial well-being after surgery have been reported in the adult gynecomastia population.^7,8^

To date, the impact of adolescent gynecomastia surgery has only been assessed using generic quality of life measures.^6,9^ While helpful in measuring general concepts of quality of life, generic patient-reported outcome measures (PROM) may lack the sensitivity to capture the nuanced issues of living with gynecomastia or chest differences.^10^ Accurate and thorough characterization of the impact of gynecomastia surgery on adolescent patients’ quality of life can aid our understanding of the significance of this surgery for this patient population, which in turn may have important implications for destigmatizing gynecomastia surgery. To this end, the present study aims to assess adolescent gynecomastia patients’ postoperative quality of life using a PROM specific for body contouring procedures. This study will also aim to identify patient and operative characteristics associated with worse quality of life scores, as well as to identify patients who may be at higher risk of low postoperative satisfaction, and for whom additional psychosocial support may be needed.

## Methods

### Study Design

This was a cross-sectional study of patients who previously underwent surgery for adolescent gynecomastia. This project was approved by the Univeristy of British Columbia Children's and Women's Research Ethics Board (H23-01006). This study is reported in accordance with the Strengthening the Reporting of Observational Studies in Epidemiology (STROBE) guidelines^11^ (Supplemental Digital Content 1).

Patients were recruited from the senior author's (JA) clinical practice at BC Children's Hospital, a quaternary hospital in Vancouver, Canada. In general, patients were considered appropriate surgical candidates if their gynecomastia had persisted for more than 2 years, and they were believed to have reached a plateau in their linear growth. Patients who underwent surgical treatment for Simon grade I, IIa, or IIb adolescent gynecomastia from May 2009 to November 2022 were eligible for inclusion in the present study. Patients with grade III gynecomastia were excluded from the present study due to low case numbers, and given that they are often treated with a surgical technique that necessitates a different type of incision and results in more significant scarring. Eligible patients were invited to participate in the study via post mail and telephone call, and informed consent was obtained.

To assess the quality of life, patients who consented to participate in the study completed the *Satisfaction with Chest* and *Psychological Function* scales of the Body Contouring Questionnaire (BODY-Q), either on an online platform or over the phone, as per participants’ preference. The BODY-Q is a PROM developed by Klassen et al^12^ for assessing patient perceptions after weight loss and/or body contouring procedures. The BODY-Q consists of 29 independently functioning scales which can be individually administered to meet the specific objectives of a given research project. In the present study, the *Satisfaction with Chest* and *Psychological Function* BODY-Q scales were administered to participants to capture both their satisfaction with the physical appearance of their chest, and their psychological well-being. Both scales consist of ten items which the patient rates on a 4-point scale, with answer options ranging from “very dissatisfied (1 point)” to “very satisfied (4 points),” or “strongly disagree (1 point)” to “strongly agree (4 points).” Higher scores on these scales reflect a better outcome.

Demographic, disease, and treatment characteristics were collected for those participants who additionally consented to chart review. For each patient, the latest documented Simon grade and weight and height measurements prior to the surgery date were recorded, where available. Where a definitive Simon grade was not documented, pre-operative surgery day photographs were reviewed by the senior author (JA), and a Simon grade was assigned retroactively. The Simon classification system^13^ was used in the present study as it is the most common classification system reported in the gynecomastia literature.^14^ Furthermore, it is the classification system that has been used in the assessment of gynecomastia patients in the senior author's clinical practice, and was thus the classification available in the medical records used to collect patient data for this study.

### Surgical Technique

All patients underwent gynecomastia surgery under general anesthesia in a standard operating theatre. Direct excision of fibroglandular and fatty breast tissue proceeded via a periareolar incision. This technique does not involve the use of liposuction to remove additional adipose tissue. Following the excision of the breast tissue, the skin was closed, and a chest binder was applied. Patients were admitted for a one-night stay in hospital. Surgical drains were not routinely used in this population. After surgery, patients were advised to wear a tight compression shirt, and to refrain from physical activity and heavy lifting for one month.

### Data Analysis

Descriptive statistics were used to summarize demographic, disease, and treatment characteristics for the study sample. Data are presented as medians (with interquartile range [IQR]), or proportions, as appropriate.

Raw scores from the BODY-Q scales were summed and converted into Rasch transformed scores (scale of 0–100) using conversion tables obtained from the authors of the BODY-Q. Mean and median scores were calculated for each scale of the BODY-Q, and the distribution of scores in the study sample was illustrated with box and whisker plots.

An independent samples *t* test was conducted to compare the mean BODY-Q scores of the study sample to previously published normative BODY-Q scores of unaffected males of a similar age and BMI range.^15^ Given the nonnormal distribution of data, linear univariate regression, Kruskall-Wallis test, and Mann-Whitney U test were used, where appropriate, to examine the association between BODY-Q *Satisfaction with Chest* or *Psychological Function* scores and the following independent variables: age at the time of survey completion; BMI at the time of survey completion; pre-operative Simon grade; whether or not the patient had postoperative complications; and whether or not the patient had revision procedures. Results are presented as *P*-values with 95% confidence intervals (CI). Statistical significance was set to *P* < .05. All statistical analyses were conducted in RStudio Version 1.4.1717 (RStudio; Boston, MA).

## Results

### Study Participants

Based on inclusion criteria, 79 patients were eligible for inclusion in the present study. Seventy-six patients were able to be contacted via telephone or post mail, and 37 consented to complete the BODY-Q (response rate = 48.7%). Twenty-three of these patients additionally consented to chart review. A flowchart outlining patient recruitment is illustrated in Supplemental Digital Content 2.

Table 1 summarizes the demographic, gynecomastia severity, and treatment characteristics of the study sample. At the time of survey completion, the median age of participants was 23.3 years, and the median BMI was 26.5. The majority (59.5%) of patients had Simon grade IIb gynecomastia. Of the 23 patients who consented to chart review, all had idiopathic adolescent gynecomastia. Furthermore, 22 of 23 patients (95.7%) endorsed a history of bullying, teasing, or feeling self-conscious about their chest appearance. The patients consenting to participate in the present study did not differ significantly from those who declined participation with respect to age, Simon grade, and follow-up duration (Supplemental Digital Content 3).

**Table 1.** Patient and Treatment Characteristics.

Characteristic	(*n *= 37)
Age at survey completion (years), median [IQR]	23.3 [20.4-26.4]
BMI at survey completion (kg/m^2^), median [IQR]	26.5 [22.5-29.0]
Age at surgery (years), median [IQR]	17 [16.1-17.6]
BMI at surgery (kg/m^2^), median [IQR]^a^	24.8 [21.5-27.6]
Follow-up duration (years), median [IQR]	7 [3.8-8.9]
Simon grade, n (%)	
I	5 (13.5)
IIa	10 (27.0)
IIb	22 (59.5)
Laterality, n (%)	
Unilateral gynecomastia	10 (27.0)
Bilateral gynecomastia	27 (73.0)
Resection weight (g) per breast, median [IQR]^b^	
Unilateral gynecomastia	61.9 [43.2-81.5]
Bilateral gynecomastia	97.0 [35.2-153.9]
Complications, n (%)^b^	
Hematoma (minor)	2 (8.7)
Seroma	7 (30.4)
No. of revision procedures, n (%)^b^	
0	17 (73.9)
1	6 (26.1)

*Note: Follow-up duration* refers to the time from surgery to survey completion. BMI, body mass index; IQR, interquartile range.

^a^ Data available for only 19 of the 23 patients consenting to chart review.

^b^ Data available for only the 23 patients consenting to chart review.

### Satisfaction and Quality of Life

**Figure 1.**
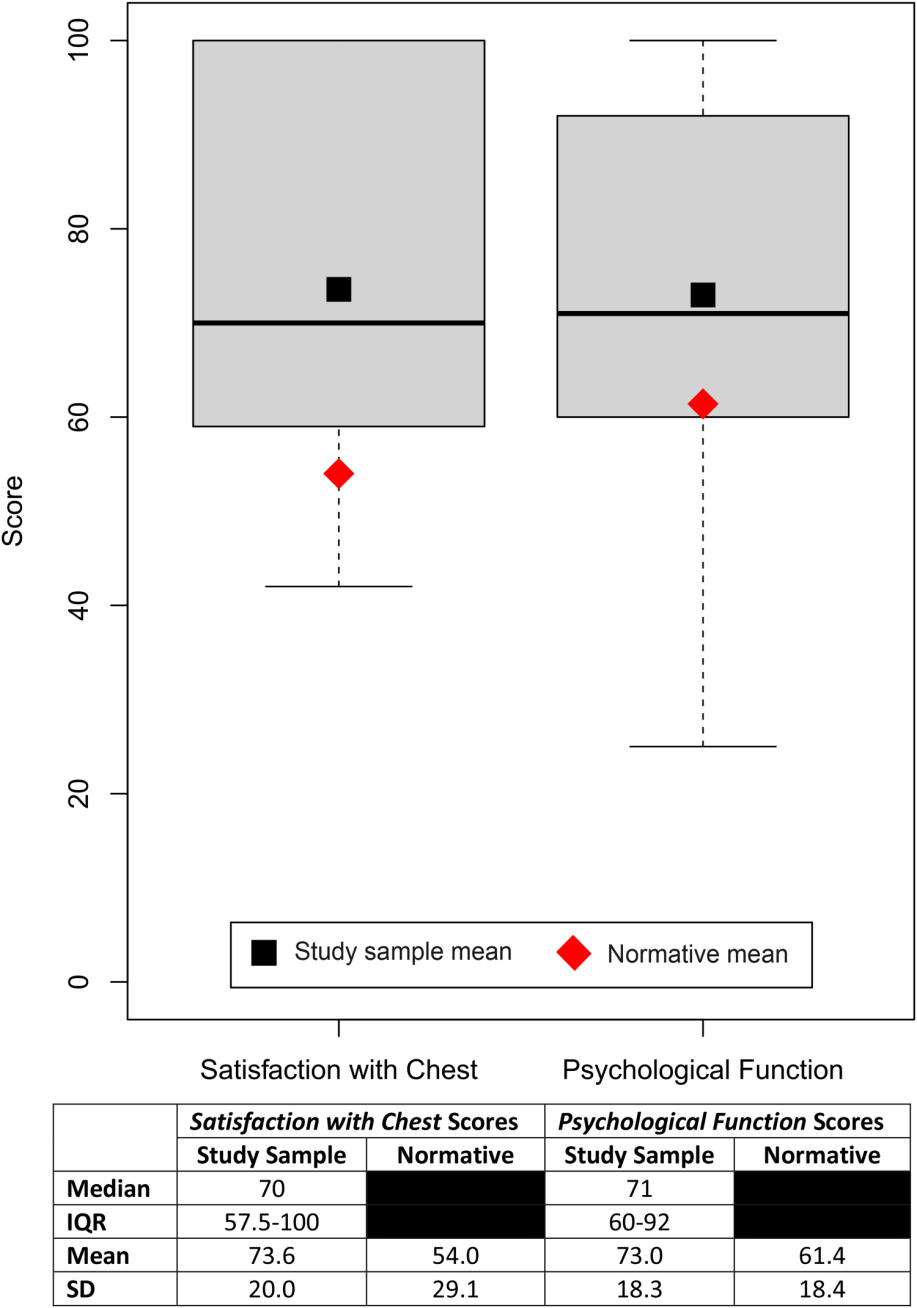
Body Contouring Questionnaire (BODY-Q) score summary.

The median scores on the BODY-Q *Satisfaction with Chest* and *Psychological Function* scales were 70 [IQR, 57.5-100] and 71 [IQR, 60-92], respectively (Figure 1). The mean score on the *Satisfaction with Chest* scale in the study sample was significantly higher than that in the normative population (73.6 and 54.0,^15^ respectively; *t*(106) = 3.7, *P* = .0004). Similarly, the mean score on the *Psychological Function* scale was significantly higher in the study sample compared to the normative population (73.0 and 61.4,^15^ respectively; *t*(132) = 3.3, *P* = .0014).

### Factors Associated with Satisfaction and Quality of Life

**Figure 2.**
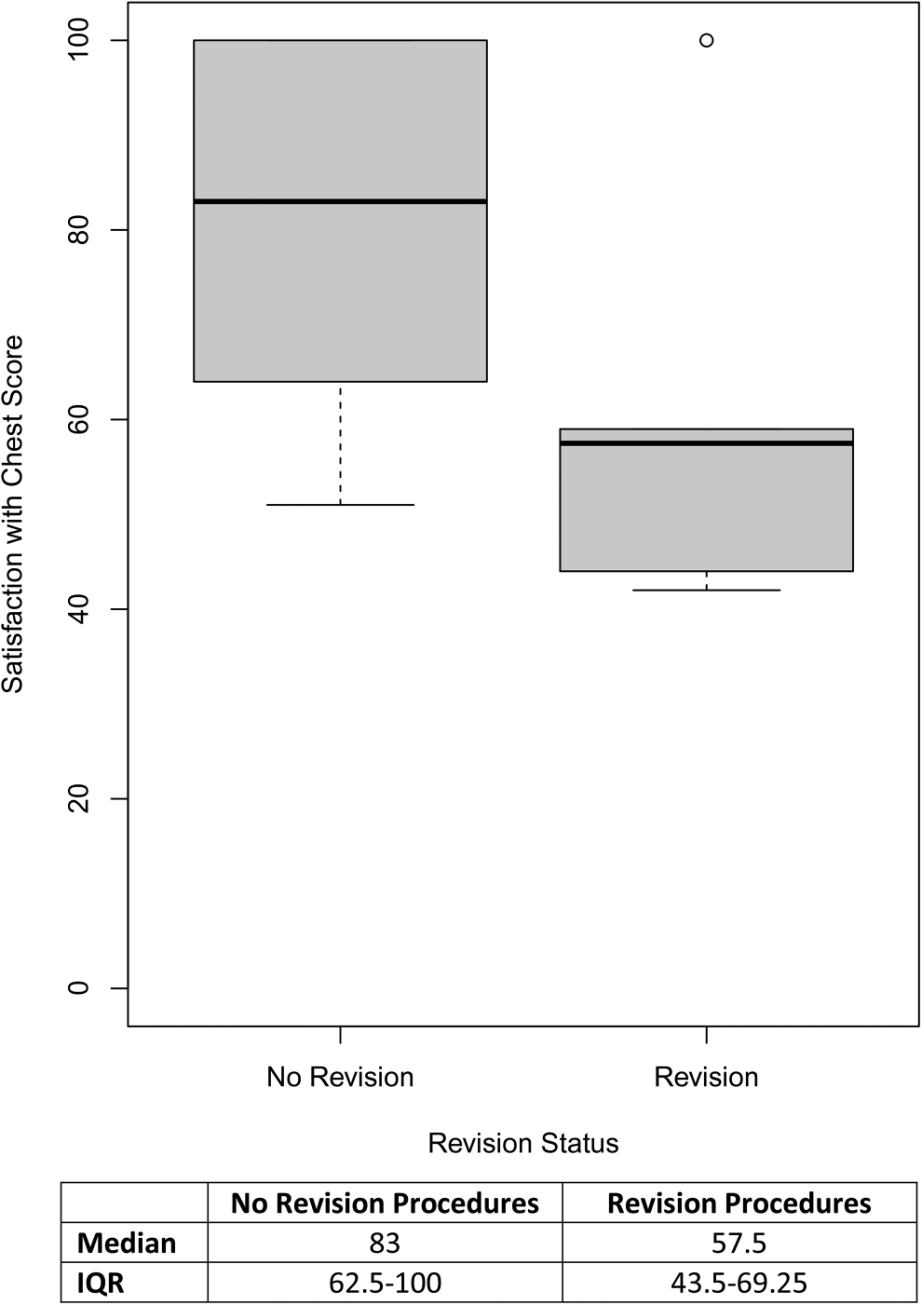
*Satisfaction with chest* scores by revision procedure status.

Patients who underwent a revision procedure had a median *Satisfaction with Chest* score 25.5 points lower than those who did not undergo a revision (Mann-Whitney *U*-test; *P* = .045, 95% CI, $3.8\times 10^{-5}$ - $4.1\times 10^{1}$ ). Figure 2 illustrates the distribution of *Satisfaction with Chest* scores in patients who underwent revision procedures versus those who did not. Revision procedures were not significantly associated with *Psychological Function* scores (*P* = .33; 95% CI, −10.0-29.0). Patients who underwent a revision procedure did not significantly differ from those who did not with respect to age and body mass index (BMI) at the time of survey completion, age, and BMI at the time of surgery, Simon grade, gynecomastia laterality, resection weight or postoperative complication status (Supplemental Digital Content 4).

Univariate linear regression did not demonstrate *Satisfaction with Chest* scores to be significantly associated with age at survey completion, *R*^2 ^= 0.034; *F*(1, 35) = 1.2; *P* = .28, nor BMI at survey completion, *R*^2 ^= 0.037; *F*(1, 35) = 1.4; *P* = .25. *Psychological Function* scores were also not associated with age at survey completion, *R*^2 ^= 0.069; *F*(1, 35) = 2.6; *P* = .12 nor with BMI at survey completion, *R*^2 ^= 0.022; *F*(1, 35) = 0.78; *P* = .38. A Kruskal-Wallis test was performed and did not reveal Simon grade to be a significant predictor of neither *Satisfaction with Chest*, H(2) = 2.7; *P* = .26, nor *Psychological Function,* H(2) = 2.2; *P* = .34 scores. Finally, a Mann-Whitney U-test showed that the presence of postoperative complication(s) was not significantly associated with *Satisfaction with Chest* (*P* = 1; 95% CI, −17.0-30.0) or *Psychological Function* (*P* = .51; 95% CI, −25.0-16.0) scores.

## Discussion

To our knowledge, the present study is the first to assess the quality of life in adolescent gynecomastia patients with a PROM specific for body contouring procedures. The BODY-Q *Satisfaction with Chest* scale captures the nuanced aspects of living with a chest difference, thus allowing for a more sensitive and accurate characterization of patients’ thoughts and feelings posttreatment. Mean BODY-Q *Satisfaction with Chest* and *Psychological Function* scores were higher by 19.6 and 11.6 points, respectively, in our study sample compared to normative scores from unaffected males of a similar age and BMI (Figure 1).

At present, the only available data on minimally important differences (MID) for the BODY-Q comes from a study in patients undergoing body contouring after weight loss surgery, in which the MID was determined to be 4.5.^16^ By this standard, the differences in scores between our study sample and the normative population are considered clinically significant, although it should be cautioned that this MID may not be reflective of the gynecomastia population. Future research to establish a MID for the BODY-Q in this patient population is needed.

While we cannot make definitive conclusions regarding the reason for higher chest satisfaction scores in our study sample compared to the normative population, we speculate that gynecomastia surgery may confer gains in chest-related quality of life that extend beyond simply addressing the perceived physical deficits experienced by adolescent males with gynecomastia. In a study by Mundy et al,^17^ researchers compared normative BREAST-Q scores to pre- and postoperative scores from a sample of women who had undergone breast augmentation. Pre-operatively, patients reported *Satisfaction with Breast* scores that were significantly lower than normative values, while postoperative scores were significantly higher than in the normative sample. The researchers suggested that breast augmentation surgery may not only correct perceived deficits, but also results in psychological benefits that enhance patients’ quality of life.^17^ Similarly, in gynecomastia patients, who are known to have impaired psychosocial functioning as a result of gender-incongruent chest development, gynecomastia surgery may do more than simply give them a perceived “normal-appearing” male chest—it additionally imparts psychological benefits which in turn may enhance one's satisfaction with their appearance and body image. Alternatively, the higher chest satisfaction scores in our study sample may be explained by the Hawthorne effect, which posits that research participants may improve their behavior as a result of their awareness of being studied.^18^

*Satisfaction with Chest* scores were comparable to postoperative scores reported in the adult gynecomastia literature.^8,19,20^ Satisfaction with chest appearance was found to be significantly lower in those patients who underwent a revision procedure after the primary gynecomastia surgery. This is consistent with several other reports of decreased satisfaction among patients who required revision surgery versus those who did not, across a variety of surgery types.^21–23^ We speculate that this difference in satisfaction scores may be the result of unmet expectations, compounded by the added burden of undergoing additional treatment. Given the relatively high rate of revision procedures after primary gynecomastia surgery,^24^ this finding has important implications for preoperative counseling, and management of patient expectations. Surgeons should have this discussion pre-operatively, especially with patients at higher risk of requiring a revision procedure. Revision rates have previously been shown to be associated with gynecomastia severity, with higher rates reported in patients with more severe gynecomastia.^24^ While we did not find significant differences in Simon grade between patients who underwent revision surgery and those who did not in our study sample, this may be due to the small sample size and the relatively low number of revision events.

Patients’ satisfaction with chest appearance was not associated with age, BMI, Simon grade, or postoperative complication status. Furthermore, none of the assessed patient, disease, and treatment factors were significantly associated with BODY-Q *Psychological Function* scores. This is consistent with existing gynecomastia literature demonstrating gains in health-related quality of life postgynecomastia surgery, irrespective of BMI, gynecomastia severity, and complication status.^9^

Gynecomastia and its surgical treatment remain stigmatized in the medical community and society at large.^25^ There has been relatively little research dedicated to studying gynecomastia and its impact on patients, and gynecomastia treatment is often seen solely as a cosmetic procedure.^25^ This is in stark contrast to the abundance of research on breast surgery in females, and the successful medicalization of breast reconstruction, augmentation, and reduction for female patients.^25,26^ In addition to the stigma, gynecomastia patients also face financial barriers in accessing treatment. Rasko et al^27^ found that only 14 of the 61 US insurance companies evaluated in their 2019 study considered coverage for gynecomastia surgery for adolescent patients. In British Columbia, our provincial health insurance provides coverage for gynecomastia surgery for both adolescent and adult patients, thus removing this financial barrier. The relatively high satisfaction and psychological function scores seen in our sample demonstrate that patients fare well after undergoing surgical treatment for adolescent gynecomastia, which in turn may be a positive step towards the destigmatization of this procedure, understanding its significance for the psychosocial well-being of this patient population, and increasing awareness and access to surgical treatment.

### Limitations

Patients were recruited from the clinical practice of a single surgeon at a quaternary center, thus limiting the generalizability of these results. The exclusion of Simon grade III gynecomastia patients further limits the scope of our findings. The relatively small sample size (n = 37) and a response rate of 48.7% may limit the reproducibility of these findings and increase variability in the final results. However, it should be noted that a response rate of 40% is generally considered adequate,^28^ and those who agreed to participate did not differ from those who did not participate with respect to age, Simon grade, and follow-up duration. A further limitation is that the lack of preoperative quality of life scores does not allow us to directly assess the impact of gynecomastia surgery. Future research should employ a prospective cohort design and assess quality of life both before and after treatment, so as to more directly assess the impact of gynecomastia surgery on patients. Changes in scores should be compared against established clinically important differences for BODY-Q scores.

## Conclusion

Patients who underwent surgery for adolescent gynecomastia with a periareolar incision to directly excise fibroglandular and fatty breast tissue fare well with respect to satisfaction with chest appearance, and psychological function. Surgeons may consider performing surgery on appropriate adolescent candidates, as there was no difference in outcomes based on age, BMI, gynecomastia severity, or complication status. Revision procedures may result in lower postoperative satisfaction with chest appearance, and this should be an important point of discussion in pre-operative counseling and expectation management, particularly in patients with more severe gynecomastia, which has previously been shown to be associated with higher revision rates. Our findings demonstrate a positive patient-reported impact of gynecomastia surgery among our survey respondents, and this is an important step towards increased awareness, access, and acceptance of gynecomastia treatment in adolescents. Future research should measure quality of life before and after surgery, to allow for more definitive conclusions about the salutary effects of gynecomastia surgery.

## Supplemental Material

sj-docx-1-psg-10.1177_22925503241249753 - Supplemental material for Quality of Life Measured Using the BODY-Q After Adolescent Gynecomastia Surgery: A Cross-Sectional Analysis Supplemental material, sj-docx-1-psg-10.1177_22925503241249753 for Quality of Life Measured Using the BODY-Q After Adolescent Gynecomastia Surgery: A Cross-Sectional Analysis by Marta Karpinski, Young Ji Tuen, Rebecca Courtemanche and Jugpal S. Arneja in Plastic Surgery

sj-docx-2-psg-10.1177_22925503241249753 - Supplemental material for Quality of Life Measured Using the BODY-Q After Adolescent Gynecomastia Surgery: A Cross-Sectional Analysis Supplemental material, sj-docx-2-psg-10.1177_22925503241249753 for Quality of Life Measured Using the BODY-Q After Adolescent Gynecomastia Surgery: A Cross-Sectional Analysis by Marta Karpinski, Young Ji Tuen, Rebecca Courtemanche and Jugpal S. Arneja in Plastic Surgery

sj-docx-3-psg-10.1177_22925503241249753 - Supplemental material for Quality of Life Measured Using the BODY-Q After Adolescent Gynecomastia Surgery: A Cross-Sectional Analysis Supplemental material, sj-docx-3-psg-10.1177_22925503241249753 for Quality of Life Measured Using the BODY-Q After Adolescent Gynecomastia Surgery: A Cross-Sectional Analysis by Marta Karpinski, Young Ji Tuen, Rebecca Courtemanche and Jugpal S. Arneja in Plastic Surgery

sj-docx-4-psg-10.1177_22925503241249753 - Supplemental material for Quality of Life Measured Using the BODY-Q After Adolescent Gynecomastia Surgery: A Cross-Sectional Analysis Supplemental material, sj-docx-4-psg-10.1177_22925503241249753 for Quality of Life Measured Using the BODY-Q After Adolescent Gynecomastia Surgery: A Cross-Sectional Analysis by Marta Karpinski, Young Ji Tuen, Rebecca Courtemanche and Jugpal S. Arneja in Plastic Surgery
