# Supplementary material for: Quality of Life Measured Using the BODY-Q After Adolescent Gynecomastia Surgery: A Cross-Sectional Analysis
Source: Plast Surg (Oakv). 2024 May 7;33(4):601–8. doi: 10.1177/22925503241249753 (PMC11561927; doi:10.1177/22925503241249753)
Supplement: sj-docx-2-psg-10.1177_22925503241249753 - Supplemental material for Quality of Life Measured Using the BODY-Q After Adolescent Gynecomastia Surgery: A Cross-Sectional Analysis [file sj-docx-2-psg-10.1177_22925503241249753.docx]

**Supplemental Digital Content 2: Patient Recruitment Flowchart**

39 patients declined to participate

Reasons for declining were ‘not interested’.

37 patients consented to complete BODY-Q

23 patients consented to chart review

3 patients unable to contact

31 patients ineligible (Simon grade III gynecomastia)

79 patients eligible (Simon grade I, IIa, or IIb gynecomastia)

76 patients/families contacted

110 gynecomastia patients undergoing surgery (May 2009 – November 2022)
