# Supplementary material for: Quality of Life Measured Using the BODY-Q After Adolescent Gynecomastia Surgery: A Cross-Sectional Analysis
Source: Plast Surg (Oakv). 2024 May 7;33(4):601–8. doi: 10.1177/22925503241249753 (PMC11561927; doi:10.1177/22925503241249753)
Supplement: sj-docx-3-psg-10.1177_22925503241249753 - Supplemental material for Quality of Life Measured Using the BODY-Q After Adolescent Gynecomastia Surgery: A Cross-Sectional Analysis [file sj-docx-3-psg-10.1177_22925503241249753.docx]

**Supplemental Digital Content 3: Characteristics of Patients who Consented vs. Declined Study Participation**

| Characteristic | Consented  (n=37) | Declined  (n=39) | p-value |
| --- | --- | --- | --- |
| Current age (y), median [IQR] | 23.3 [20.1-26.4] | 24.8 [21.6-28.3] | 0.06‡ |
| Age at surgery (y), median [IQR] | 17.0 [16.0-17.7] | 16.8 [15.8-17.6] | 0.33‡ |
| Simon grade, n (%)  *I*  *IIa*  *IIb* | 5 (13.5)  10 (27.0)  22 (59.5) | 5 (12.8)  13 (33.3)  21 (53.8) | 0.83§ |
| Time since surgery (y), median [IQR] | 7.0 [3.8-9.0] | 7.5 [4.5-12.1] | 0.08‡ |

‡Mann-Whitney U test

§Chi-square test
