# Supplementary material for: Quality of Life Measured Using the BODY-Q After Adolescent Gynecomastia Surgery: A Cross-Sectional Analysis
Source: Plast Surg (Oakv). 2024 May 7;33(4):601–8. doi: 10.1177/22925503241249753 (PMC11561927; doi:10.1177/22925503241249753)
Supplement: sj-docx-4-psg-10.1177_22925503241249753 - Supplemental material for Quality of Life Measured Using the BODY-Q After Adolescent Gynecomastia Surgery: A Cross-Sectional Analysis [file sj-docx-4-psg-10.1177_22925503241249753.docx]

**Supplemental Digital Content 4: Characteristics of Patients who Underwent Revision Surgery vs. No Revision Surgery**

| Characteristic | Revision (n=6) | No Revision (n=17) | p-value |
| --- | --- | --- | --- |
| Age at survey completion (y), median [IQR] | 21.8 [19.1-26.7] | 23.2 [20.8-24.0] | 0.92‡ |
| BMI at survey completion (kg/m^2^), median [IQR] | 27.0 [23.1-28.9] | 24.0 [22.1-27.5] | 0.81‡ |
| Age at surgery (y), median [IQR] | 17.0 [16.7-17.2] | 17.2 [16.1-17.9] | 0.55‡ |
| BMI at surgery (kg/m^2^), median [IQR]* | 24.8 [24.1-29.4] | 25.2 [20.8-27.0] | 0.49‡ |
| Follow up duration (y), median [IQR] | 4.8 [2.1-9.6] | 5.33 [4.3-7.3] | 0.75‡ |
| Simon grade, n (%)  *I*  *IIa*  *IIb* | 0 (0)  3 (50)  3 (50) | 4 (23.5)  4 (23.5)  9 (52.9) | 0.30§ |
| Laterality, n (%)  *Unilateral gynecomastia*  *Bilateral gynecomastia* | 1 (16.7)  5 (83.3) | 5 (29.4)  12 (70.6) | 0.54§ |
| Resection weight (g) per breast, median [IQR]† | 120.6 [92.6-163.2] | 52.8 [29.5-118] | 0.17‡ |
| Complications, n (%)†  *Yes*  *No* | 3 (50)  3 (50) | 6 (35.3)  11 (64.7) | 0.53§ |

*Data available for only 19 of the 23 patients consenting to chart review

**†**Data available for only the 23 patients consenting to chart review

‡Mann-Whitney U test

§Chi-square test

**Note:** *Follow-up duration* refers to time from surgery to survey completion.
